# Disentangling the Consequences of Latino Immigrants’ Unauthorized Status for the Health of Their U.S.-Born Children

**DOI:** 10.1007/s40615-025-02447-0

**Published:** 2025-04-25

**Authors:** Tianjian Lai

**Affiliations:** Department of Medicine and Center for Health and Social Sciences, The University of Chicago, 5841 S. Maryland Avenue, MC 1005, Suite M200, Chicago, IL 60637 USA

**Keywords:** Immigration, Health, Children, Legal status, Unauthorized/undocumented

## Abstract

Immigrants’ legal status has been described as a social determinant of health that shapes health outcomes through multiple channels. Furthermore, legal status matters not only for immigrants themselves, but also for their family members, including their U.S.-born citizen children. Nonetheless, few studies have empirically disentangled the possible mediating mechanisms through which parents’ legal status shapes their children’s health. This paper applies path analysis to data from the 2014–2019 California Health Interview Survey to examine how Latino immigrant parents’ unauthorized status impacts the health of their U.S.-born citizen children. I consider how the effects of parents’ unauthorized status for their children’s health are mediated by children’s healthcare access, household food insecurity, family income, and parents’ own physical and mental health. I find that parents’ unauthorized status has a negative but indirect effect on their children’s health through increasing household risk of poverty and food insecurity. These results have significant implications for the well-being of millions of children in the United States who have legally vulnerable parents.

**Supplementary Information:**

The online version contains supplementary material available at 10.1007/s40615-025-02447-0.

## Introduction

The 11 million unauthorized immigrants in the United States are parents to over 5 million children, 4.4 million of whom are U.S.-born citizens [1]. Parents’ undocumented status has significant implications for the health of their children, even if their children possess US citizenship. For instance, previous studies have uncovered how U.S.-born children living in mixed legal status families report worse self- and parent-rated health relative to the children of U.S.-citizen parents [2, 3]. Other studies have found that U.S.-born Latino adolescents with unauthorized immigrant parents or who worry about the deportation of their family members report negative health metrics, such as high blood pressure, allostatic load, and anxiety [4, 5].

Immigration and health scholars have pointed to immigrants’ legal status as a social determinant of health that affects health outcomes through multiple mechanisms, such that “removing any one proximate cause… would not eliminate the relationship between social conditions and disease given the existence of other proximate causes” [6, p.19]. Furthermore, these scholars have hypothesized that the effects of immigrants’ unauthorized status may spill over even for the health outcomes of those who are not legally vulnerable themselves, including unauthorized immigrants’ U.S.-born citizen children [7]. Nonetheless, while past studies have theorized that immigrant parents’ legal status may impact their children’s well-being through multiple proximate mechanisms, few studies have empirically modeled these mediating channels.

This paper analyzes data from the California Health Interview Survey to understand *how* parents’ legal status may affect their children’s health. I focus on outcomes among Latino children of immigrants, who make up nearly a quarter of all children living in the United States [8]. Through applying path analysis, I disentangle the mechanisms through which parents’ legal vulnerability shapes their U.S.-born children’s health outcomes. I consider the mediating role of family income, children’s access to timely healthcare, household food insecurity, and parents’ own physical and mental health, with significant implications for policy interventions that support the health and development of the children of immigrants.

### How Immigrant Parents’ Legal Status Shapes Their Children’s Health

The harmful effects of vulnerable legal status impact not only immigrants themselves, but also their children, who grow up in households navigating the cumulative risk factors of financial instability, lack of access to resources, and parenting strain [9]. The literature on legal status and child well-being has identified several important pathways through which parents’ legal status may shape the health outcomes of their children. First, unauthorized status significantly constrains the employment opportunities of adult immigrants, reducing family income and limiting the financial resources available to their children [10, 11]. In addition, parents’ vulnerable legal status may intersect with economic hardship to limit children’s access to health services, increase household food insecurity, and negatively affect parents’ own physical and mental health [5, 12–14]. Together, financial hardship, diminished access to healthcare and health-promoting resources, and poor parental health may serve as pathways through which parents’ vulnerable legal status impacts their children’s health.

Unauthorized immigrants in the United States face poor employment conditions, particularly following the passage of the 1986 Immigration Reform and Control Act (IRCA) which aimed to curtail the employment of unauthorized immigrants and greatly increased the sanctions that employers faced for hiring undocumented workers [15]. Likewise, these immigrants are particularly vulnerable to wage and labor violations, as lack of legal status may prevent migrants from turning to authorities for help when facing exploitation by employers [16, 17]. Furthermore, fear of apprehension and deportation, as well as state-level policies barring unauthorized immigrants from obtaining drivers’ licenses, may further restrict their employment opportunities. As a result, relative to documented immigrants, undocumented immigrants may concentrate in low-wage, socially isolated jobs that have little potential for upward mobility and few healthcare benefits such as employer-sponsored health plans [10, 18]. In addition, unauthorized immigrant parents may be ineligible for benefits that may promote health and well-being among their citizen children. For instance, families in which the heads of household do not have a social security number cannot receive an Earned Income Tax Credit (EITC) subsidy for low-income families, regardless of their child’s citizenship status.

Such barriers to household economic well-being have significant implications for the financial conditions under which the children of legally vulnerable immigrants grow up, with an estimated 32% of the citizen children of unauthorized immigrant parents living in poverty (compared to 10% of children of U.S. citizens) and 75% of children with unauthorized immigrant parents living in households below 185% of the federal poverty level [19, 20]. Growing up in a socioeconomically disadvantaged household is highly predictive of poor health among the children of immigrants [11, 21]. As such, household poverty may be one mechanism through which parents’ legal vulnerability structures children’s health outcomes.

Parents’ precarious legal and financial status may together reduce their children’s access to healthcare and health-promoting resources, contributing to children’s negative health outcomes. Punitive immigration policies targeting unauthorized immigrants may have spillover effects for the public service usage of those who are not themselves unauthorized, including U.S. citizens [22]. In 2018, the Trump administration proposed amendments to the public charge rule that added programs such as the Supplemental Nutrition Assistance Program (SNAP) and Medicaid to the list of public programs that would disqualify enrolled immigrants from receiving a visa or green card. Although this amendment was only in effect between February 2020 and March 2021, it resulted in significant disenrollment from public programs. Following the 2018 announcement, 20% of adults in immigrant families across the United States reported not enrolling in government benefit programs for fear of becoming ineligible for a green card [23]. This disenrollment rate was particularly high for families with children (17%) compared to without (9%), and for Latino adults (21%) compared to whites (9%) [23].

Due to their legal vulnerability and uncertainty over program eligibility, unauthorized immigrant parents may decline to enroll their children in public health services even when their child may be eligible, with negative implications for their children’s access to healthcare [24–27]. Vargas [28] finds that children living in mixed legal status families were significantly less likely to be enrolled in Medicaid in districts with high deportation risk. Nationally, children in low-income, mixed legal status families are also significantly more likely to be uninsured relative to the children of U.S. citizens [29]. Low rates of insurance or Medicaid coverage and high rates of household poverty may translate into reduced healthcare access among the children of legally vulnerable parents. For instance, Yun et al. [30] found that the children of previously undocumented parents were more likely to have delayed access to preventive care. Thus, parents’ unauthorized immigration status may negatively shape children’s health outcomes through diminished access to care.

Similarly, reduced family income and disenrollment from or ineligibility for public services may promote food insecurity among mixed legal status families. Children living in immigrant families, particularly with parents who lack documentation status, report decreased enrollment in supplemental food programs such as SNAP and the Special Supplemental Program for Women, Infants, and Children (WIC) when living in areas with high deportation risk, anti-immigrant rhetoric, or following the 2020 amendment to the public charge rule [31, 32]. Furthermore, while U.S.-citizen children are eligible for SNAP regardless of their parents’ legal status, the amount of assistance that families receive may be greatly impacted by the legal status of other household members. A family of four U.S. citizens would receive $512 in SNAP benefits per month, while a family of four with two unauthorized immigrant parents would only receive $160 per month [33]. As such, past studies have found high rates of food insecurity among noncitizen and unauthorized immigrant households, and increased rates of food insecurity for mixed status households in the context of punitive immigration policies such as 287(g) [5, 34, 35]. Food insecurity has significant negative effects on children’s health, with participation in programs such as SNAP found to promote children’s health through improving access to food [36, 37]. Thus, household food insecurity may be another mechanism through which parents’ vulnerable legal status shapes their children’s outcomes.

Furthermore, immigrant parents’ vulnerable legal status and financial insecurities may be detrimental for their own physical and mental health. The daily stress of poor working conditions, financial insecurity, fear of deportation, stigma, and uncertainty tied to unauthorized status negatively impacts the mental health of legally vulnerable Latino immigrants [38–40]. Undocumented parents in particular report psychological distress stemming the threat of family separation and feelings of disempowerment due to their vulnerable legal status [14, 41]. Additionally, while legally vulnerable immigrants may enter the United States with comparable or better physical health outcomes relative to the U.S. born, their health statuses may deteriorate with longer time spent in the United States and more exposure to reduced healthcare access, high rates of poverty, and stress stemming from their legal status [42–44]. The stress and poor health faced by unauthorized immigrant parents may have negative effects on their parenting practices, decreasing their availability and responsiveness to their children’s needs and damaging their relationships with family members [45, 46]. Given the deleterious effects of vulnerable legal status for immigrants’ mental and physical health, and the negative implications of poor parental health for their children’s outcomes, parents’ unauthorized status may also negatively affect children’s health through depressing parents’ own physical and mental health statuses.

### Latino Children of Immigrants in California

Latinos are the largest ethnic minority group in the United States, making up over 60 million residents and 19% of the U.S. population [47]. An estimated one-third of Latinos are immigrants and 13% are unauthorized [48]. Furthermore, over 25% of all Latino children in the United States have at least one parent who is undocumented, making them highly susceptible to the vulnerabilities posed by their parents’ unauthorized immigration status [49].

As the state with the largest immigrant population in the country, California is a highly significant case for examining how immigrant parents’ legal status shapes the health of their children. Nearly half of children in California have at least one foreign-born parent, and over 10% of California children have a parent who is undocumented [50]. Among children of immigrants in California, 60% are Latino [50]. These children frequently grow up in households struggling with financial precarity. One in four Latino children in California live in poverty, and the median household income of Latinos in California is $16,000 below the state median [51]. Furthermore, Latinos in California report high rates of food insecurity, with Latino immigrants who do not have permanent residency more vulnerable to food insecurity relative to permanent residents and naturalized citizens [35].

Despite financial vulnerabilities, Latino immigrants in California are eligible for certain health services unavailable in other state contexts. For instance, children in California who meet certain household income thresholds are eligible for Medi-Cal, California’s public health insurance program, regardless of their parents’ or their own legal status. In 2024, California became to first state to expand public insurance access to all low-income immigrants regardless of legal status. Nonetheless, vulnerable immigration status may still endanger the well-being of immigrants and their family members. For instance, 44% of immigrant parents in California who lack a green card reported avoiding government benefits due to fear of being deemed a “public charge” and being disqualified from obtaining permanent residency in the future [50].

## Methods

### Data and Measures

Data for this study comes from the California Health Interview Survey (CHIS) adult and child samples. CHIS is a representative telephone survey of households in California conducted by the UCLA Center for Health Policy Research, covering adults (age 18 +), adolescents (ages 12–17), and children (ages 0–11). CHIS is uniquely suited for studying the effects of parents’ legal status on children’s health outcomes. It contains detailed information on both mothers’ and fathers’ citizenship and lawful permanent residency (LPR) status, children’s health conditions and healthcare access, household financial status and food insecurity, and parents’ physical and mental health. I merged CHIS adult samples, which contain information on parents’ health and household demographic information, with child samples, which contain information on parents’ legal status, children’s health outcomes, and children’s healthcare utilization. CHIS imputes missing data using the hot-deck method, which replaces missing data with observations from most proximate cases.

To hold constant children’s citizenship and nativity status, I limited this study to U.S.-born Latino children with at least one parent born outside of the United States. I aggregated data from the 2014–2019 CHIS surveys, applying weights according to CHIS specifications. I restricted the sample to children ages 4 to 11, with a final sample of 1841 children. Adolescents aged 12–17 in CHIS self-reported their general physical health, while the parents of children under 12 reported on their children’s health. Given the differences in the parent- and self-reported nature of the child health measure, I was unable to concatenate the two samples.

My outcome of interest for this study is child’s *general physical health condition*. Parents rated their children as having excellent, very good, good, fair, or poor health. I coded this variable on a 1–5 scale, with 1 representing poor health and 5 representing excellent health. My main predictor of interest is *parents’ unauthorized immigration status*, coded as 1 if the immigrant parent did not report having a green card, and 0 if the parent reported being a U.S. citizen or lawful permanent resident. Following past research on the effects of parents’ legal status on children, I classified parents’ unauthorized immigration status according to the parent with the most vulnerable status [3]. While CHIS contains legal status information for both mothers and fathers, only the responding parent of the CHIS adult questionnaire reported on their physical and mental health. Thus, the legal status of the adult questionnaire respondent may not always align with the low-status anchoring classification used to categorize parents’ legal status. Nonetheless, the mental and physical health of a citizen or permanent resident parent may be affected by their co-parent’s legal vulnerability, given the stress experienced by documented members in mixed legal status households [40]. Sensitivity analyses conducted using only children whose parents are of the same legal status yielded comparable results (Supplemental Tables 1 and 2).

I consider the mediating role of five variables to examine the effects of parents’ legal status on their children’s health outcomes: household income, children’s timely access to healthcare, parents’ emotional well-being, parents’ physical health, and household food security. *Household income* is measured as a ratio of family income to the federal poverty level. Children’s *timely access to healthcare* is coded as a dichotomous variable: 1 if the child did not report any delays to needed healthcare in the past 12 months, and 0 if the child reported any delays. *Household food security* is a dichotomous variable, coded as 1 if the child lived in a household that did not experience food insecurity, and 0 if the child lived in a food-insecure household. Food-insecure households were labeled as such if parents reported that the food in the household did not last and the household could not afford to buy more, or if household members could not afford to eat balanced meals, cut or skipped meals, ate less than they should, or went hungry but did not eat due to lack of money to buy food. I also examined the mediating effects of *parents’ physical health*, a dichotomous variable where 1 represents “very good” or “excellent” health, and 0 represents “good,” “fair,” or “poor” health. *Parents’ mental well-being* is coded as a dichotomous variable, with 1 representing a score of 7 or lower on the Kessler Psychological Distress Scale, and 0 representing a score of 8 or higher (an indicator of psychological distress). Finally, I control for parent and child demographics, including child’s age and dichotomous variables measuring whether the child is female, and whether the parent who responded to the CHIS survey ever obtained a college degree or spoke English “very well” or “well” (rather than “not well,” or “not at all”).

### Analytic Plan

**Fig. 1:**
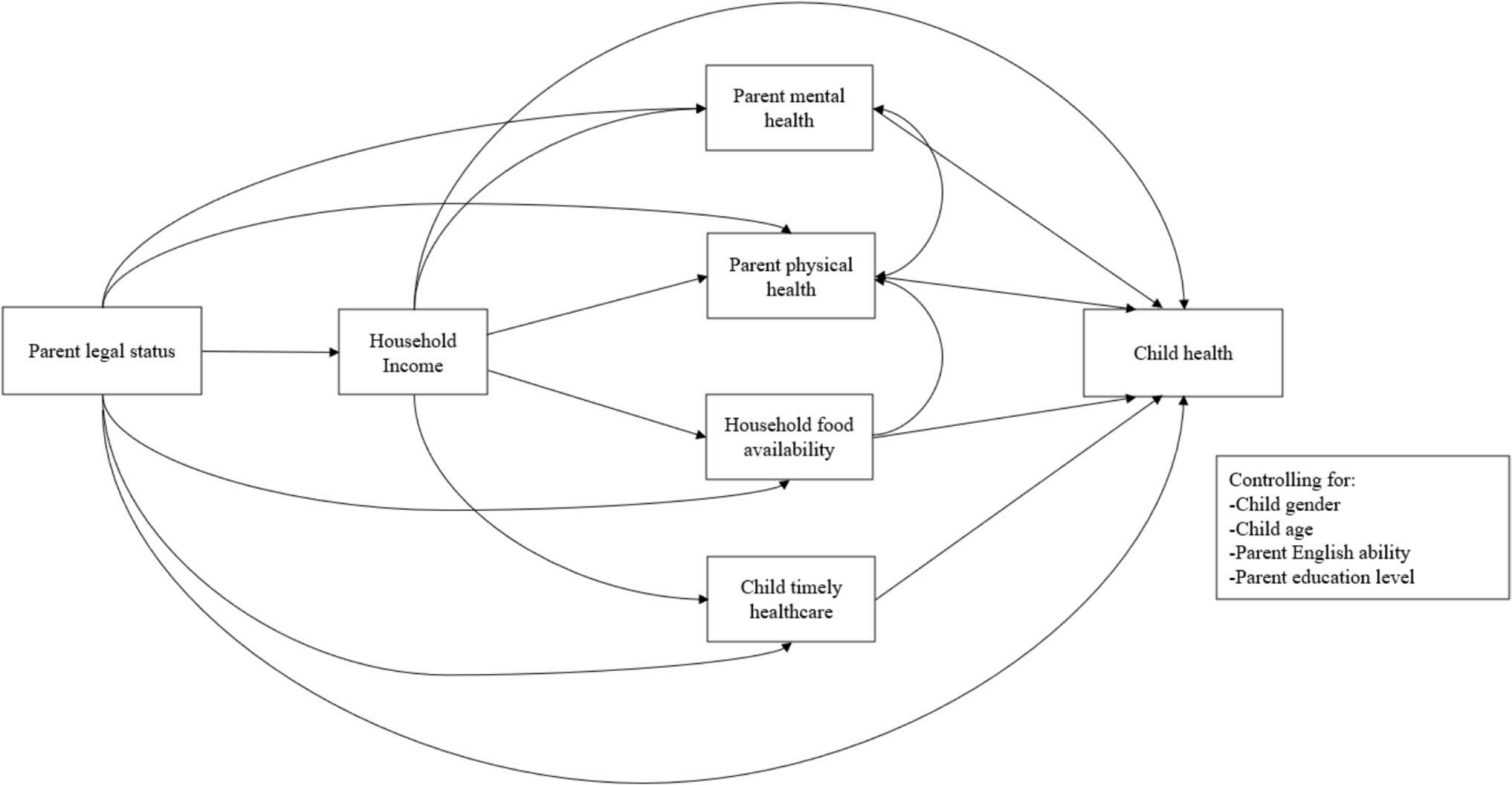
Decomposing paths through which parents’ legal status shapes their children’s health

I employ path analysis to decompose the mediating pathways through which parents’ legal status affects their children’s health outcomes. Using path analysis allows me to simultaneously estimate the direct and indirect effects of parents’ legal status on children’s health and examine interrelationships between independent variables. Figure 1 presents the paths I delineate for my path analysis model. I hypothesize that parents’ unauthorized status will negatively affect their children’s health through several mediating mechanisms, namely, by decreasing household income, negatively affecting parents’ mental and physical health, and diminishing household food availability and children’s access to timely healthcare. In addition, I hypothesize that low household income exacerbated by parents’ unauthorized status will have an additional negative effect on children’s health by reducing children’s access to healthcare, decreasing food security, and harming parents’ physical and mental health. I evaluate model fit using the standardized root mean squared residual (SRMR), which averages the standardized residuals between the observed and hypothesized covariance matrices.

## Results

Table 1 presents descriptive statistics for the sample. Differences between the children of documented and undocumented immigrants were evaluated using chi-squared tests. The U.S.-born children of unauthorized immigrant parents report a significantly lower general health status score relative to the U.S.-born children of documented immigrant parents (3.9 vs. 4.2). They also live in households with a significantly lower household income as a percentage of the federal poverty level (1.1 vs. 2.4) and lower rates of food security (54.4% vs. 75.8%). Furthermore, unauthorized immigrant parents are significantly less likely to be college-educated (5.3% vs. 23.8%), to speak English “well” or “very well” (33.1% vs. 69.9%), and to report “excellent” or “very good” health (28.3% vs. 38.9%). Children’s access to timely medical care and parents’ degree of mental distress do not differ significantly by parents’ legal status.

**Table 1:** Descriptive statistics

	Documented parent (51.3%)	Undocumented parent (48.7%)
Child		
General health scale	**4.2**	**3.9**
Receives timely healthcare	98.0%	96.7%
Female	53.2%	51.3%
Age	7.6	7.5
Household		
Household income relative to FPL	**2.4**	**1.1**
Household food security	**75.8%**	**54.4%**
Parent		
Mental distress	12.0%	10.7%
Very good/excellent physical health	**38.9%**	**28.2%**
Speaks English well/very well	**69.8%**	**33.1%**
College degree	**24.8%**	**5.3%**

**Boldface** indicates statistically significant difference (*p* < 0.05) between the children of documented and undocumented parents (chi-squared test)

Next, I employed path analysis to disentangle the direct (Table 2) and indirect (Table 3) effects of parents’ legal status on children’s health outcomes. I found that among the U.S.-born children of Latino immigrants, the effect of parents’ unauthorized status on health is mediated by household poverty and food insecurity. Net of controls, parents’ unauthorized status has a significant negative indirect effect on their children’s health but does not exert a statistically significant direct effect on health. Parents’ unauthorized status has a significant negative effect on household income, while unauthorized status and low household income together have direct negative effects on household food security. Children’s health is directly and negatively affected by low household income and household food insecurity. Younger children, girls, and children whose parents speak English well also reported better health outcomes relative to older children, boys, and children whose parents have limited English proficiency. Furthermore, parents’ English proficiency is associated with both significantly positive direct and indirect effects on child health, while parents’ college education has a marginally positive direct effect and significantly positive indirect effect on child health. The SRMR value (0.024) indicates good model fit.

**Table 2:** Direct effects from structural equation model

	Coefficient	SE
Parent’s undocumented status on…		
Child health	0.009	0.072
Child access to timely care	− 0.019	0.013
Household income	− 0.724***	0.105
Household food security	− 0.112**	0.041
Parent mental well-being	0.014	0.028
Parent physical health	− 0.024	0.039
Household income on…		
Child health	0.027**	0.011
Child access to timely care	0.002	0.002
Household food security	0.037***	0.010
Parent mental well-being	0.001	0.007
Parent physical health	0.006	0.007
Household food availability on…		
Child health	0.228**	0.077
Parent physical health	0.131**	0.380
Parent physical health on…		
Child health	0.421***	0.070
Parent mental well-being on…		
Child health	− 0.011	0.123
Child access to timely care on		
Child health	0.194	0.182
Parent speaks English well on…		
Child health	0.306***	0.079
Child access to timely care	− 0.020	0.013
Household income	0.731***	0.103
Household food security	0.079 +	0.042
Parent mental well-being	0.006	0.028
Parent physical health	0.174***	0.041
Parent college education on…		
Child health	0.168 +	0.089
Child access to timely care	− 0.013	0.019
Household income	1.985***	0.329
Household food security	− 0.013	0.039
Parent mental well-being	− 0.011	0.041
Parent physical health	0.054	0.062
Child age on…		
Child health	− 0.027*	0.013
Female child on…		
Child health	0.152*	0.068

+*p* < 0.10

^*^p < 0.05

^**^p < 0.01

^***^p < 0.001

**Table 3:** Indirect effects from structural equation model

	Coefficient	SE
Parent’s undocumented status on…		
Child health	− 0.068**	0.023
Child access to timely care	− 0.001	0.001
Household food security	− 0.027***	0.007
Parent mental well-being	− 0.001	0.005
Parent physical health	− 0.004	0.005
Household income on…		
Child health	0.011*	0.004
Parent physical health	0.005*	0.002
Food security on…		
Child health	0.055	0.019
Parent speaks English well on…		
Child health	0.116***	0.024
Child access to timely care	0.001	0.001
Household food security	0.027***	0.008
Parent mental well-being	0.001	0.005
Parent physical health	0.004	0.005
Parent college education on…		
Child health	0.126***	0.036
Child access to timely care	0.004	0.003
Household food security	0.073***	0.016
Parent mental well-being	0.003	0.013
Parent physical health	0.012	0.014

+*p* < 0.10

^*^p < 0.05

^**^p < 0.01

^***^p < 0.001

## Discussion

### Conclusion

Immigrants’ vulnerable legal status may shape not only their own outcomes, but also those of their U.S. citizen children. While past studies have theorized that immigrant parents’ legal status may impact their children’s well-being through multiple proximate causes including socioeconomic status, access to services, and parents’ own health, few studies have empirically disentangled the direct and indirect effects of parents’ legal status on their children’s health. This paper examines *how* parents’ legal status may affect their children’s health outcomes by disentangling the potential mediating roles of financial precarity, children’s access to healthcare, household food insecurity, and parents’ physical and mental health on the relationship between parents’ legal status and their children’s health. I applied path analysis to uncover the mechanisms through which parents’ legal status influences their children’s health, finding that the effects of parents’ unauthorized status on their children’s health are mediated by household poverty and food insecurity.

My findings that the health outcomes of U.S.-born children with undocumented parents are significantly shaped by household poverty and food insecurity have significant policy implications. First, unauthorized immigrants face tremendous barriers to their socioeconomic mobility. They are not authorized to work legally in the United States, and as a result, are barred from formal employment and paths to socioeconomic attainment. Providing a path to work authorization for legally vulnerable immigrants would result in significant increases in income and decreases in poverty levels among immigrant families [19]. Giving unauthorized immigrants a pathway to legal employment and improving the conditions under which they work would have tremendous positive implications for their children, whose health outcomes are affected not only directly by household poverty, but also indirectly by the negative effects of household poverty on household food insecurity.

Furthermore, this study contributes to research illustrating food insecurities experienced by legally vulnerable families. I find that Latino parents’ unauthorized status reduces their household’s access to food, with significant negative implications for their children’s health. Public charge designations hinder immigrants who are enrolled in public services from obtaining lawful permanent residency, including the 2018 rule which penalized immigrants’ access to SNAP. Eliminating these designations would improve access to health-promoting resources for both unauthorized immigrants and their U.S.-born children, who may be eligible for these benefits but fail to enroll due to their vulnerable parents’ fears of being “on the radar” of immigration enforcement [27, 52]. Furthermore, unauthorized immigrant parents may be misinformed or unaware of their U.S. citizen children’s right to public service access. Outreach programs that keep immigrant parents informed of their U.S.-citizen children’s rights to assistance would further improve U.S.-born children’s access to resources beneficial for their health.

Finally, while we find that parents’ vulnerable legal status exerts a significant *indirect* effect on their children’s health through multiple mechanisms, we did not find a statistically significant *direct* effect of parents’ undocumented status on their children’s health. This finding may illustrate the protective effects of citizenship for the U.S.-born children of unauthorized immigrant parents, which entitles children to certain services and safeguards despite their parents’ unauthorized immigration status. Underscoring the protective effects of citizenship for children’s health outcomes is especially pertinent given recent political efforts to do away with birthright citizenship for the children of unauthorized immigrants in the United States. On January 20, 2025, Donald Trump issued an executive order to end birthright citizenship for children born in the United States to parents who are not citizens or lawful permanent residents. Terminating birthright citizenship for the U.S.-born children of unauthorized immigrants is likely to have significant adverse effects for children’s health outcomes and exacerbate their vulnerability to their parents’ precarious legal status.

While children’s own citizenship may offer some protection for their health outcomes, citizenship nonetheless does not fully shield children of immigrants from the detrimental consequences of their parents’ unauthorized status. Immigrants’ legal status shapes the resources, environment, and opportunities that they can mobilize for the benefit of their families. This paper shows that parents’ unauthorized status engenders multiple causes of poor health among their U.S.-born children. Addressing these causes individually would be ineffective at fully bridging gaps in health outcomes between the children of undocumented and documented immigrant parents, as parents’ vulnerable status invariably structures additional avenues of stratification. Establishing a pathway to legalization for unauthorized immigrant parents would fundamentally improve the outcomes of their children, over four million of whom are U.S.-born citizens.

### Limitations

California is an especially significant state for studying the effects of parents’ unauthorized status, with one in ten children in the state having at least one parent who is unauthorized [50]. California has relatively tolerant policies towards unauthorized immigration and full-scope public insurance coverage for low-income residents [53]. Low-income children in California are eligible for Medicaid regardless of their or their parents’ immigration status, resulting in high rates of public insurance coverage. Three in 5 Latino children in California are covered by Medicaid, compared to 43% of children in the state and 36% nationally [51, 54]. Furthermore, in 2024, California became the first state to extend Medicaid coverage to unauthorized immigrants, facilitating healthcare access for legally vulnerable parents and positively affecting the health outcomes of children of immigrants [55].

Thus, findings based on the immigrant population in California may not be representative of the United States as a whole. For instance, studies on the children of immigrants in California have produced more optimistic findings regarding the healthcare access of children living in mixed status families. These studies have found that the U.S.-born children of undocumented parents living in California have comparable rates of healthcare access and insurance coverage relative to the citizen children of documented parents [56–58]. Likewise, this study found that U.S.-citizen children of unauthorized immigrants do not differ significantly from the children of documented immigrants on their access to timely healthcare.

By contrast, studies conducted in states such as Texas or nationally have found that the U.S.-born children of unauthorized immigrants experience diminished access to healthcare, particularly under contexts of unfavorable policies toward immigrants [25, 28, 29]. Similarly, studies have found greater incidence of poor mental and physical health among Latino children residing in states with hostile sentiment and policies towards immigrants [59]. Nonetheless, these findings from California that immigrant parents’ legal status shapes their children’s health through multiple mechanisms are likely to be echoed or amplified in areas with higher levels of anti-immigrant sentiment and policy. Future research should examine how parents’ legal status structures the healthcare access and health outcomes of children living in different contexts.

Finally, while this study is among the first to disentangle *how* parents’ legal status shapes their children’s health, it does not exhaustively explore all the complex mediating factors that explain the relationship between immigration status and health. Applying path analysis allows me to disentangle direct and indirect factors impacting the health of children of immigrants. Nonetheless, because path analysis simultaneously estimates multiple relationships between endogenous and exogenous variables, each new variable adds many additional parameters. As in other linear models, additional parameters increase the risks of identification issues, non-convergence, lower statistical power, and multicollinearity. Future research (particularly studies with greater statistical power) should further examine how additional factors, including parents’ and children’s perceptions of discrimination, may influence the relationship between parents’ immigration status and their children’s health.

## Supplementary Information

Below is the link to the electronic supplementary material.

Supplementary file1 (DOCX 21 KB)

## Data Availability

This research utilizes confidential data from the California Health Interview Survey, which is available by application to the UCLA Center for Health Policy Research.
