# Supplementary material for: Disentangling the Consequences of Latino Immigrants’ Unauthorized Status for the Health of Their U.S.-Born Children
Source: J Racial Ethn Health Disparities. 2025 Apr 25;13(4):2663–72. doi: 10.1007/s40615-025-02447-0 (PMC13346281; doi:10.1007/s40615-025-02447-0)
Supplement: Supplementary file 1 — Supplementary file1 (DOCX 21 KB) [file 40615_2025_2447_MOESM1_ESM.docx]

**Supplemental Tables**

**Supplemental Table 1:** Sensitivity analyses: Direct effects from structural equation model (parents of same status only)

|  | Coefficient | SE |
| --- | --- | --- |
| Parent's undocumented status on… |  |  |
| Child health | 0.167 | 0.125 |
| Child access to timely care | -0.012 | 0.016 |
| Household income | -0.667*** | 0.185 |
| Household food security | -0.041 | 0.067 |
| Parent mental wellbeing | 0.034 | 0.046 |
| Parent physical health | -0.028 | 0.067 |
|  |  |  |
| Household income on… |  |  |
| Child health | 0.080* | 0.034 |
| Child access to timely care | 0.002 | 0.002 |
| Household food security | 0.068** | 0.021 |
| Parent mental wellbeing | -0.046* | 0.019 |
| Parent physical health | -0.034 | 0.020 |
|  |  |  |
| Household food availability on… |  |  |
| Child health | 0.146 | 0.116 |
| Parent physical health | 0.083 | 0.057 |
|  |  |  |
| Parent physical health on… |  |  |
| Child health | 0.343** | 0.121 |
|  |  |  |
| Parent mental health on… |  |  |
| Child health | -0.011 | 0.218 |
|  |  |  |
| Child access to timely care on |  |  |
| Child health | 0.257 | 0.426 |
|  |  |  |
| Parent speaks English well on… |  |  |
| Child health | 0.386** | 0.136 |
| Child access to timely care | -0.017 | 0.018 |
| Household income | 0.704** | 0.234 |
| Household food security | 0.107 | 0.071 |
| Parent mental wellbeing | 0.084* | 0.038 |
| Parent physical health | 0.223** | 0.075 |
|  |  |  |
| Parent college education on… |  |  |
| Child health | 0.179 | 0.181 |
| Child access to timely care | 0.011 | 0.010 |
| Household income | 0.785 | 0.495 |
| Household food security | 0.169* | 0.080 |
| Parent mental wellbeing | -0.036 | 0.081 |
| Parent physical health | 0.030 | 0.119 |
|  |  |  |
| Child age on… |  |  |
| Child health | -0.028 | 0.024 |
|  |  |  |
| Female child on… |  |  |
| Child health | 0.097 | 0.107 |

+ p < 0.10

* p < 0.05

** p < 0.01

*** p < 0.001

**Supplemental Table 2:** Sensitivity analyses: Indirect effects from structural equation model (parents of same status only)

|  | Coefficient | SE |
| --- | --- | --- |
| Parent's undocumented status on… |  |  |
| Child health | -0.075* | 0.036 |
| Child access to timely care | -0.002 | 0.002 |
| Household food security | -0.045*** | 0.013 |
| Parent mental wellbeing | 0.030+ | 0.018 |
| Parent physical health | 0.016 | 0.015 |
|  |  |  |
| Household income on… |  |  |
| Child health | 0.001 | 0.016 |
| Parent physical health | 0.006 | 0.004 |
|  |  |  |
| Food security on… |  |  |
| Child health | 0.029 | 0.022 |
|  |  |  |
| Parent speaks English well on… |  |  |
| Child health | 0.147** | 0.045 |
| Child access to timely care | 0.002 | 0.002 |
| Household food security | 0.048** | 0.014 |
| Parent mental wellbeing | -0.032 | 0.020 |
| Parent physical health | -0.011 | 0.018 |
|  |  |  |
| Parent college education on… |  |  |
| Child health | 0.107* | 0.060 |
| Child access to timely care | 0.002 | 0.002 |
| Household food security | 0.053 | 0.040 |
| Parent mental wellbeing | -0.036 | 0.030 |
| Parent physical health | -0.008 | 0.027 |

+ p < 0.10

* p < 0.05

** p < 0.01

*** p < 0.001
